# Editorial: Encouraging health research productivity in complex humanitarian crises: Somalia

**DOI:** 10.3389/fpubh.2024.1377036

**Published:** 2024-02-20

**Authors:** Abdi Gele, Amina Jama Mahmud, Bernadette Nirmal Kumar, Cynthia Khamala Wangamati, Hodan A. Duale, Mamunur Rahman Malik, Mekdes Gebremariam, Ragnhild Dybdahl

**Affiliations:** ^1^Division for Health Services, Norwegian Institute of Public Health (NIPH), Oslo, Norway; ^2^Department of Medical Sciences, Faculty of Medicine, Uppsala University, Uppsala, Sweden; ^3^Division for Health Services, University of Oslo, Oslo, Norway; ^4^Institute of Health and Society, Somali Institute for Health Research (SIHR), Garowe, Puntland, Somalia; ^5^Department of Maternal Health, World Health Organization, Mogadishu, Somalia

**Keywords:** Somalia, humanitarian crisis, health research, enabling research production, Fregile

An estimated two billion people are living under conditions of armed conflict around the globe, and it is estimated that one in every 45 people require humanitarian assistance. The impacts of these crises on the health of the affected population is severe (1); hence, evidence-based health solutions are crucial in these contexts. Health research is essential for guiding policies and programmes to improve access to health services, and to inform and scale interventions that build community capacity to mitigate the devastating effects of humanitarian crises. However, with limited resources, basic necessities often become a priority for countries, leaving health research as a secondary consideration (2). Somalia is ranked as one of the poorest countries in the world and has had more than three decades of armed conflict. As a result, the country has limited institutional capacity and weak health research systems, mainly due to intrinsic challenges such as lack of research infrastructure, meager human resource for research and lack of research trainings in academic institutions (3).

In response to the challenges above, we launched a call for papers on health in humanitarian crises with a particular focus on Somalia (Figure 1) in early 2023. The aim of the Research Topic was to promote research production in Somalia, which is an urgent national and global imperative. We made the scope of the Research Topic broad to cover all topics within the health field, with special emphasis on areas of national priority.

**Figure 1:**
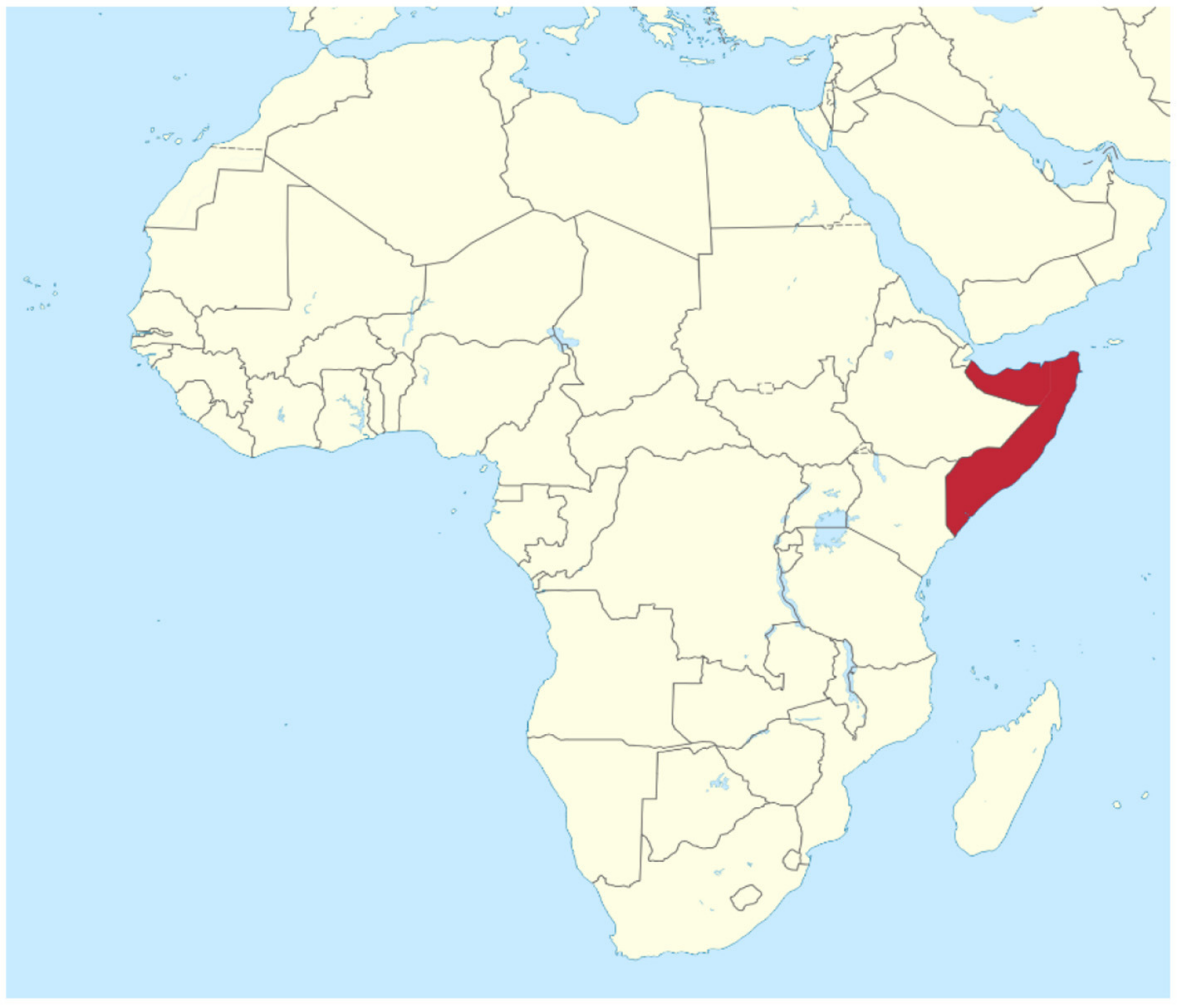
Country affiliation of authors contributing to the Research Topic. Reprinted with permission from *Somalia in Africa (-mini map -rivers).svg* by TUBS published 7 April 2011, licensed under CC BY-SA 3.0, Wikimedia Commons: https://commons.m.wikimedia.org/wiki/File:Somalia_in_Africa_(-mini_map_-rivers).svg.

In total, seven papers addressing diverse health challenges facing Somalia were accepted. Two papers investigated the challenges facing health research; Ahmed et al. reported on the lack of national health research institutions and health research policy in the country. The existing health research projects either received limited funding from international organizations or were self-funded by the researchers. The authors highlighted the importance of the development of a national health research policy and priorities, allocation of sufficient and sustainable funding, capacity building of staff and strengthening of the national health research governance. Ssendagire et al. also examined health research priorities which, if implemented, can inform local interventions required to accelerate progress toward universal health coverage in Somalia. The study developed unique health research questions which were categorized under health systems, services, and social determinants, communicable diseases, non-communicable diseases and reproductive, maternal, new-born, child, adolescent health and nutrition. Across the four categories, an overall list of questions with the highest priority scores was developed to inform health research resources to questions that contribute to generation of local health system knowledge.

Focusing on maternal health, Dualeh et al. explored the maternal health services available for pastoralists in Somalia. Somalia has one of the highest maternal mortality rates in the world (693\100,000). Pastoralists, who constitute 26–30% of the Somali population (4), have virtually no access to maternal healthcare. The authors described the constraints that hamper the efficiency of the health facilities that serve pastoralist communities and suggested that affordable maternal healthcare can be achieved by including pastoralists' healthcare in national health priorities.

Humanitarian efforts in conflict-afflicted regions in Somalia rarely address the mental health challenges of the population. Salad et al. investigated the prevalence of mental disorders and psychological trauma among conflict- affected population in Southern Somalia. The overall prevalence of common mental disorders was 78.1%, which is among the highest in the world. Authors underscored the urgent need to integrate mental health and psychosocial support within the primary healthcare and other service sectors such as education.

Hassan et al.'s study on Urinary tract infections (UTIs) among diabetes patients in Mogadishu and sensitivity to drugs showed high prevalence, with Escherichia coli being the most common organism. All isolates were found to be resistant to a number of antibiotics. The most sensitive antibiotics were colistin (99.6%), imipenem (88.6%) and gentamycin (70%). Mohamed et al. examined the prevalence and antimicrobial resistance of Uropathogenic E. coli (UPEC) in pregnant women. Out of the 220 urine samples, 42 (19%) were positive. The authors suggested concrete actions to prevent transmission of resistant pathogens and complications in both pregnant mothers and the unborn baby.

Finally, Dirie et al., investigated the prevalence of urolithiasis and their demographic and computer tomography (CT) characteristics among subjects under CT scans in Mogadishu. A CT scan-based urolithiasis prevalence of 14.8% was found. Considering the high prevalence of the disease, authors underscored the need to invest more in prevention and treatment facilities while also training urologists that are capable of utilizing minimally invasive techniques.

There are many topics not covered in this Research Topic and more research on gender, children, and translation of knowledge into practice would have been valuable. In addition to the lack of resources in health research, conducting research in humanitarian crises settings poses numerous ethical challenges. These challenges are addressed to a limited degree in this issue and deserve further attention. Despite these limitations, this collection of papers illustrates that solid and interesting research with implications for policy and practice, and that adds to the global knowledge base, can be done, and is being done, in one of the most challenging humanitarian contexts of the world.

## Author contributions

AG: Writing—original draft, Writing—review & editing. AJ: Writing—review & editing. BK: Writing—review & editing. CW: Writing—review & editing. HD: Writing—review & editing. MM: Writing—review & editing. MG: Writing—review & editing. RD: Writing—review & editing.
